# Factors associated with falls in hemodialysis patients: a case-control study

**DOI:** 10.1590/1518-8345.5300.3505

**Published:** 2021-11-19

**Authors:** Ignacio Perez-Gurbindo, Ana María Álvarez-Méndez, Rafael Pérez-García, Patricia Arribas-Cobo, María Teresa Angulo Carrere

**Affiliations:** 1 Universidad Complutense de Madrid, Facultad de Enfermería, Fisioterapia y Podología, Madrid, Madrid, Spain.; 2 Hospital Universitario Infanta Leonor, Servicio de Nefrología, Madrid, Madrid, Spain.

**Keywords:** Postural Balance, Accidental Falls, Renal Dialysis, Risk Factors, Body Weight Changes, Clinical Nursing Research, Equilibrio Postural, Accidentes por Caídas, Diálisis Renal, Factores de Riesgo, Cambios en el Peso Corporal, Investigación en Enfermería Clínica, Equilíbrio Postural, Acidentes por Queda, Diálise Renal, Fatores de Risco, Alterações no Peso Corporal, Pesquisa em Enfermagem Clínica

## Abstract

**Objective:**

to identify possible associations between a higher probability of falls among hemodialysis patients and laboratory values, comorbidities, pharmacological treatment, hemodynamic changes, dialysis results and stabilometric alterations.

**Method:**

this was a retrospective case-control study with hemodialysis patients. Patients in a hemodialysis unit who had suffered one or more falls were included in the case group. Patients from the same unit who had not suffered falls were the controls. Data were gathered from the patients’ clinical history and also from the results of a balance test conducted six months before the study.

**Results:**

thirty-one patients were included (10 cases and 21 controls). Intradialytic body weight change was significantly greater among cases (p <0.05). Patients in the case group also presented greater lateral instability after dialysis (p <0.05). Other factors such as high blood pressure, antihypertensives, beta-blockers, and lower heart rates were also associated with falls.

**Conclusion:**

a greater intradialytic weight change was associated with an increase in risk of falls. Nursing staff can control these factors to prevent the incidence of falls in dialysis patients.

## Abstract (es)

**Objetivo:**

identificar la posible asociación entre los valores analíticos, comorbilidades, tratamiento farmacológico, cambios hemodinámicos, resultado de la diálisis y alteraciones estabilométricas con una mayor probabilidad de caídas en pacientes en hemodiálisis.

**Método:**

estudio retrospectivo de casos y controles en pacientes en hemodiálisis. Se consideraron como casos pacientes de una unidad de hemodiálisis que habían sufrido una o varias caídas. Los controles fueron pacientes de la misma unidad que no sufrieron caídas. Se obtuvieron los datos de la historia clínica de los pacientes y, también, se valoró una prueba de equilibrio realizada 6 meses antes a dichos pacientes.

**Resultados:**

se incluyeron 31 pacientes (10 casos y 21 controles). El cambio de peso durante la diálisis fue significativamente mayor en el grupo de personas que sufrieron una caída (p <0,05). Los pacientes que sufrieron una caída presentaron mayor inestabilidad lateral después de la diálisis (p <0.05). Otros factores como la hipertensión arterial, los antihipertensivos, los betabloqueantes y las frecuencias cardiacas más bajas también se relacionaron con las caídas.

**Conclusión:**

un mayor cambio de peso intradiálisis se relaciona con mayor riesgo de caídas. El control de estos factores por parte del personal de enfermería podría prevenir la incidencia de caídas en pacientes dializados.

## Abstract (pt)

**Objetivo:**

identificar a possível associação entre valores laboratoriais, comorbidades, tratamento farmacológico, alterações hemodinâmicas, resultado da diálise e alterações estabilométricas com uma maior probabilidade de quedas em pacientes de hemodiálise.

**Método:**

estudo caso-controle retrospectivo em pacientes de hemodiálise. Foram analisados os casos de pacientes de uma unidade de hemodiálise que sofreram uma ou mais quedas. Os controles foram pacientes da mesma unidade que não sofreram quedas. Os dados foram obtidos a partir do histórico clínico dos pacientes e, também, de um teste de equilíbrio realizado seis meses antes nesses pacientes.

**Resultados:**

31 pacientes foram avaliados (10 casos e 21 controles). A alteração de peso durante a diálise foi significativamente maior no grupo de pessoas que sofreu uma queda (p <0,05). Pacientes que sofreram queda apresentaram maior instabilidade lateral após a diálise (p <0,05). Outros fatores, como hipertensão, anti-hipertensivos, betabloqueadores e frequência cardíaca mais baixa, também foram associados às quedas.

**Conclusão:**

uma maior alteração do peso intradialítico está relacionada a um maior risco de quedas. O controle desses fatores pela equipe de enfermagem poderia prevenir a incidência de quedas em pacientes em diálise.

## Introduction

Moderate to severe Chronic Kidney Disease (CKD) (stages 3-5) affects 6.8%-9.5% of the population^(1)^ and involves the accumulation of waste substances such as uremic toxins, which cannot be eliminated due to impaired renal function. In Latin America the prevalence of patients treated with hemodialysis (HD) is 451 *per* million inhabitants^(2)^. In these circumstances, patients must undergo dialysis several times a week, with the aim of eliminating uremic toxins and excess fluids, as well as rebalancing the concentrations of ions and other substances, which affect the body’s homeostasis. These biochemical alterations affect the functioning of organs and systems related to balance; in fact, hyponatremia, which affects 6%-29% of patients receiving HD^(3)^, has been associated with an increased risk of falls.

The excess liquid that must be extracted from patients varies depending on the weight gain that they experience in the interdialytic period and the difference between their calculated optimum weight or dry weight, which is defined as weight when there is no fluid excess or deficiency, without the presence of detectable peripheral edemas, with normal blood pressure, and with no postural hypotension. The excess volume is removed during dialysis, which lasts about 4 hours. The higher the weight gain, the higher the ultrafiltration rate required, resulting in an increased risk of hypotension during dialysis or postHD orthostatic hypotension^(4-5)^, both situations associated with greater morbidity and mortality in patients receiving HD^(6-7)^.

Therefore, HD produces hemodynamic changes and acute homeostasis, affecting postural control. Previous studies have observed that, after HD sessions, patients suffer changes in postural control^(8-9)^. Likewise, severe CKD, even when treated by HD, results in the progressive deterioration of structures involved in balance. One example is HD-related amyloidosis^(10)^, which affects joints such as the hip, which plays an important role in postural control in older adults^(11)^. In addition, patients treated with HD usually have other comorbidities, which require treatment and in many cases lead to the polymedication of HD patients, which poses a greater risk of falls^(12)^.

Consequently, numerous factors may place HD patients’ postural control at risk. Preventing falls in HD patients is essential, because the consequences in terms of quality of life, associated morbidity and reduction in life expectancy are very important^(13-14)^. The nursing staff in charge of our dialysis unit is responsible for connecting, supervising and disconnecting the dialyzed patient. In these processes, clinical situations can occur, which are already contemplated by protocols, given that greater postural instability is observed after the sessions. In turn, other subclinical situations possibly related to the factors already mentioned continue to pose a risk, which keeps the incidence of falls high among our patients, at levels similar to those observed in prevalence studies, in which the incidence was between 1-1.6 falls *per* patient-year^(15-16)^.

The aim of this study was to identify possible associations between a higher probability of falls among hemodialysis patients and the laboratory values, comorbidities, pharmacological treatment, hemodynamic changes, dialysis results and stabilometric alterations.

## Method

### Study design

This was a retrospective case-control study with a ratio of 1 case/2 controls, with hemodialysis patients. The STROBE (Strengthening the Reporting of Observational Studies in Epidemiology) guide for observational studies was followed, as recommended by the EQUATOR network.

### Study location and period

The study was conducted in the hemodialysis unit of the Infanta Leonor University Hospital (HUIL), in Madrid (Spain), between January and October 2019.

### Participants

The study was conducted with 31 patients: 10 cases and 21 controls matched by age, sex and years receiving dialysis treatment. Patients with prevalent CKD treated with HD three times a week were included and voluntarily agreed to participate in the study. Patients who presented central neurological pathologies, vestibular or visual alterations without optical correction, deformations of the locomotor system and those who could not remain standing were excluded.

Patients in the hemodialysis unit who had suffered one or more falls in the last 6 months and who reported this in the questionnaires administered monthly by the nursing staff were considered cases (n=10). Thus, the participants were unaware that the event “fall” was what categorized them as cases in the study. The controls (n=21) were patients from the same unit who did not report falls during the study period and they were also blind to this criterion. The nurses who had collected the data relative to falls were also unaware of this study.

### Data collection

After the cases were recruited, the researchers reviewed the patients’ medical records with their prior consent, using IBM’s SPSS^®^ (Statistics Package for the Social Sciences) to create a database with all the information. Laboratory values, medication, hemodynamic values during the dialysis sessions and session values obtained by the dialyzer were analyzed. We also evaluated a balance study conducted 6 months earlier in these same patients, using an AMTI AccuGait force platform, previously validated by other study^(17)^. In this study, each patient underwent a stabilometry test before (preHD) and immediately after (postHD) the same dialysis session.

### Variables

The researchers gathered the following information regarding the general characteristics of the patients: age, sex, body mass index (BMI) and years in renal replacement therapy.

Laboratory variables collected from the patients’ medical records included sodium (mEq/L-milliequivalent *per* liter), potassium (mEq/L), calcium (mg/dL-milligrams *per* deciliter), phosphorus (mg/dL) and beta-2 microglobulin protein. The values of the most recent lab tests were considered. All of them were continuous variables.

The most frequent comorbidities in patients treated with HD were diabetes, high blood pressure (HBP) and heart disease. The number of medications each patient took simultaneously was counted and categorized according to criteria already described in the variable polymedication, which was considered positive if the patient took 4 or more drugs. These were all dichotomous categorical variables.

The HD variables recorded by the dialyzer at the start of the study and included in the analysis were total ultrafiltration (UF), Kt and Kt/v. UF is the fluid removed from the blood through the dialysis membrane. Kt and Kt/v are measures based on the urea kinetic model and have been classically used to express the dialysis dose and to estimate the efficacy of the dialyzer. Kt represents urea clearance (K) multiplied by the time of HD (t) and Kt/v is Kt divided by the volume (v) of urea distribution. All these factors were continuous quantitative variables.

Hemodynamic variables collected before and after HD were also analyzed. The following were recorded: 1) systolic blood pressure (SBP), diastolic blood pressure (DBP) and pulse pressure; 2) heart rate; 3) dry weight, preHD weight and postHD weight. Changes and differences between preHD and postHD variables were calculated. All these factors were continuous quantitative variables.

The stabilometry variables studied as factors were extracted from a stabilometry study conducted with hemodialized patients 6 months prior to this study and all the patients that composed this sample were included. In this stabilometry study, the balance of patients was tested before and after HD, following the same protocol for all patients, which had been used previously in a similar study^(18)^. The tests were carried out by two people trained to handle the platform in an office prepared for this purpose, where the lighting conditions were the same throughout the day. The variables analyzed were: 1) average displacement range of the center of pressure (CoP) on the Y axis (Y range) and X axis (X range) measured in cm (centimeter); 2) maximum and average velocity (Vymax, Vxmax and Vavg) of these movements measured in cm/s (centimeter/second); and 3) the area that included the displacement of the CoP with 95% confidence (Area95) measured cm^2^ (square centimeters). All these factors were continuous quantitative variables.

### Data processing and analysis

After the database was built, it was cleansed. The Kolmogorov-Smirnov (K-S) test was used to determine whether continuous variables followed a normal distribution. The results of the continuous quantitative variables were expressed as mean ± standard deviation.

The comparison of means of the different quantitative factors between the cases and the controls was carried out using the Student’s t-test or the Mann-Whitney test, according to the distribution presented by the analyzed variables. The comparison of the frequency of the different qualitative factors in the case and control groups was performed using the chi-square test (Χ^2^) for qualitative variables. P values <0.05 were considered significant in Pearson’s test. The odds ratio (OR) was calculated, in addition to confidence intervals (CI) at 95%.

The area under the ROC (Receiver Operating Characteristic) curve was calculated to obtain the discriminatory cut-off point of the variable difference in weight between cases and controls. Youden’s J statistic was calculated to determine the optimal cut-off point^(19)^. This index is defined by sensitivity + specificity-1. Its value can range from-1 to 1 and equals zero when the test values yield the same proportion of positive results in the control group and the case group, thus rendering the test useless. A value of 1 indicates a perfect test.

Statistical analysis was performed using IBM software^®^ SPSS Statistics 15.0 Inc. Chicago, IL.

### Ethical aspects

This study was approved by the Ethics Committee (HUIL-HGUGM) with the title “Balance disorders in patients with chronic kidney disease on hemodialysis (HD)”, under protocol code: HUIL - 18/001; protocol version: 4.5 and version date: February 15, 2019.

## Results

A total of 31 patients, 19 (61.3%) men and 12 (38.7%) women, participated in the study. No significant gender differences were found among the cases (6 men and 4 women) and the controls (13 men and 8 women). Table 1 shows the differences between cases and controls in terms of age, BMI, years treated with HD, laboratory variables, comorbidities and polymedication status. Participants in the case group were 10 times less likely to be hypertensive than the control group (OR= 0.105, 95%CI=0.02-0.71). A significant difference was observed between mean levels of beta-2 microglobulin between cases and controls, with a 95% confidence level, the mean levels of cases were 0.09-9.39 higher than controls (95%CI=0.09-9.39).

**Table 1:** Differences in analytical variables and frequency of comorbidities between cases (falls) and controls (no falls) in hemodialysis patients (n=31) at the Nephrology Service of the Infanta Leonor University Hospital. Madrid, Spain, 2019

	Cases	Controls	
Age (years)	66.3 ± 11.78	71.43 ± 11.83	p*= 0.268
BMI (kg/m^2^)^†^	28.66 ± 5.62	19.36 ± 43.3	p*= 0.857
Years in dialysis treatment	10.5 ± 9.19	7.53 ± 8.02	p*= 0.374
**Analytical variables**			
Sodium (mEq/L)^‡^	138.2 ± 2.9	139.29 ± 1.9	p*= 0.22
Potassium (mEq/L)	5.42 ± 0.77	4.95 ± 0.54	p*= 0.06
Calcium (mg/dL)^§^	8.46 ± 0.38	8.58 ± 0.5	p*= 0.51
Phosphorus (mg/dL)	4.87 ± 1.74	4.43 ± 1.09	p*= 0.39
Beta-2 microglobulin	31.74 ± 5.37	27 ± 6.16	**p** * **= 0.046** *
**Comorbidities**			
Diabetes	Cases	Controls	
Yes	4 (12.9%)	9 (29%)	p*= 0.88
No	6 (19.4%)	12 (38.7%)	
HBP^||^	Case	Controls	
Yes	5 (16.1%)	19 (61.3%)	**p** * **= 0.012** *
No	5 (16.1%)	2 (6.5%)	
Heart Disease	Cases	Controls	
Yes	7 (22.6%)	8 (25.8%)	p*= 0.097
No	3 (9.7%)	13 (41.9%)	

* p = Level of significance;

† BMI = Body mass index (kg/m^2^ = Kilogram/square meter);

‡ mEq/L = Miliequivalent/liter;

§ mg/dL = Miligram/deciliter;

|| HBP = High blood pressure

Although there was no difference in the distribution of the polymedication variable between cases and controls, we analyzed possible differences in the main groups of drugs in terms of falls. The contingency tables for each group and their significance are presented in Table 2. Participants in the case group were 10 times more likely to use antihypertensive drugs than those in the control group (OR=10, 95%CI=1.63 - 61.46). Likewise, the cases were 9 times more likely to use ß2 antagonists than the controls (OR=9, 95%CI=1.55-52.27). Distribution in diuretic therapy showed significant differences, although it was not possible to calculate the odds ratio because there were no patients in the case group treated with diuretics.

**Table 2:** Frequency of polymedication and treatment with fall-risk increasing drugs among cases (falls) and controls (no falls) in patients (n=31) receiving hemodialysis at the Nephrology Service of the Infanta Leonor University Hospital. Madrid, Spain, 2019

Oral Antidiabetics	Cases	Controls	
Yes	2 (6.5%)	5 (16.1%)	p*= 0.81
No	8 (25.8%)	16 (51.6%)	
Antihypertensives	Case	Controls	
Yes	8 (25.8%)	6 (19.4%)	**p** * **= 0.007**
No	2 (6.5%)	15 (48.4%)	
Benzodiazepines	Case	Controls	
Yes	4 (12.9%)	9 (29%)	p*= 0.88
No	6 (19.4%)	12 (38.7%)	
Antidepressants	Case	Controls	
Yes	1 (3.2%)	3 (9.7%)	p*= 0.74
No	9 (29%)	18 (58.1%)	
Sedatives	Case	Controls	
Yes	2 (6.5%)	4 (12.9%)	p*= 0.95
No	8 (25.8%)	17 (54.8%)	
Antihistamines	Case	Controls	
Yes	2 (6.5%)	5 (16.1%)	p*= 0.81
No	8 (25.8%)	16 (51.6%)	
ß2 Antagonists	Case	Controls	
Yes	6 (19.4%)	3 (9.7%)	**p** * **= 0.009**
No	4 (12.9%)	18 (58.1%)	
Polymedication	Case	Controls	
Yes	1 (3.2%)	5 (16.1%)	p*= 0.363
No	9 (29%)	16 (51.6%)	

* p = Level of significance

We analyzed the distribution of HD variables as recorded by the dialysis machine, in addition to the hemodynamic values of blood pressure, heart rate and weight recorded by the nursing team before and after dialysis. Table 3 presents the results.

**Table 3:** Differences in dialysis and hemodynamic session variables between cases (falls) and controls (no falls) in hemodialysis patients (n=31) at the Nephrology Service of the Infanta Leonor University Hospital. Madrid, Spain, 2019

Hemodialysis (HD) session variables	Cases	Controls	
UF*	2801.9 ± 764.28	2384.37 ± 976.17	p^†^= 0.251
Kt^‡^	57.16 ± 4.94	58.4 ± 5.45	p^†^= 0.567
Kt/v^§^	1.79 ± 0.3	1.92 ± 0.42	p^†^= 0.375
**Hemodynamic variables**			
SBP^||^ preHD^¶^ (mmHg)	135.25 ± 20.17	139.65 ± 27.2	p^†^= 0.88
DBP** preHD^¶^ (mmHg)	70.66 ± 14.26	70.35 ± 10.97	p^†^= 0.95
SBP^||^ postHD^††^ (mmHg)	129.22 ± 27.67	138.95 ± 21.61	p^†^= 0.31
DBP** postHD^††^ (mmHg)	69.22 ± 13.53	73.1 ± 12.16	p^†^= 0.45
HR^‡‡^ preHD^¶^ (bpm^§§^)	71.6 ± 9.86	79.09 ± 13.45	p^†^= 0.13
HR^‡‡^ postHD^††^ (bpm^§§^)	72.1 ± 8.67	81.24 ± 12.27	**p** ^†^ **= 0.044** *
PreHD weight^¶^ (kg)	79.1 ± 13.82	71.62 ± 21.16	p^†^= 0.32
PostHD weight^††^ (kg)	76.76 ± 13.54	70.01 ± 20.69	p^†^= 0.36
Intradialysis weight difference (kg)^||||^	2.34 ± 0.88	1.61 ± 0.89	**p** ^†^ **= 0.042**

* UF = Ultrafiltration;

† p = Level of significance;

‡ Kt = Urea clearance multiplied by dialysis time;

§ Kt/v = Kt divided by the volume of distribution of urea;

|| SBP = Systolic blood pressure;

¶ preHD = Pre dialysis;

** DBP = Diastolic blood pressure;

†† PostHD = Post dialysis;

‡‡ HR = Heart rate;

§§ bpm = Beats *per* minute;

|||| kg = kilogram

An ROC curve was used to find the discriminatory cut-off point between cases and controls in terms of the variable of intradialytic weight change. The area under the curve was 0.721, with a CI of 95%CI (0.526-0.917). The cut-off point was set at 1.1 kg, 1.9 kg and 2.7 kg, which resulted in a sensitivity of 100%, 70% and 40%, respectively and a specificity of 28.6%, 66.7% and 95.2%, respectively. The Youden index (J=0.367) indicated that the point that determined the highest sensitivity and specificity together was 1.9 kg.

Table 4 presents the means of cases and controls in terms of the variables obtained in the balance test, performed before and after the same dialysis session. This group of variables did not present a normal distribution (K-S p<0.05), so the Mann-Whitney U test was used to assess whether there were significant differences between cases and controls.

**Table 4:** Differences produced by hemodialysis in the stabilometric variables between cases (falls) and controls (no falls) in patients (n=31) treated with hemodialysis at the Nephrology Service of the Infanta Leonor University Hospital. Madrid, Spain, 2019

Stabilometry variables		Case	Controls	
X range	PreHD*	3.58 ± 1.62	3.02 ± 1.12	p^†^= 0.091
	PostHD^‡^	4.25 ± 2.27	3.43 ± 1.74	p^†^= 0.162
Y range	PreHD*	3.76 ± 1.54	2.85 ± 1.21	**p** ^†^ **= 0.012** *
	PostHD^‡^	4.22 ± 2.41	3.43 ± 1.74	p^†^= 0.113
V x max^||^	PreHD*	11.83 ± 6.56	9.76 ± 4.69	p^†^= 0.130
	PostHD^‡^	14.53 ± 9.45	11.26 ± 5.45	p^†^= 0.336
V^||^ y max	PreHD*	14.76 ± 8.81	10.78 ± 7.93	**p** ^†^ **= 0.022** *
	PostHD^‡^	17.48 ± 13.09	12.97 ± 9.88	p^†^= 0.101
Mean V^||^	PreHD*	3.87 ± 2.32	3.13 ± 1.95	p^†^= 0.137
	PostHD^‡^	4.25 ± 2.92	3.39 ± 1.91	p^†^= 0.150
Area 95	PreHD*	8.78 ± 6.96	5.73 ± 4.25	p^†^= 0.066
	PostHD^‡^	11.65 ± 11.04	7.43 ± 6.07	p^†^= 0.308

* PreHD = Pre dialysis;

† p = Level of significance;

‡ PostHD = Post dialysis;

§ CI = Confidence interval;

|| V = Velocity

The controls presented significant differences (p<0.05) in the PreHD and PostHD data for all the stabilometric variables, except for V x max. In contrast, the differences related to HD among the cases were not significant (p>0.05) for any of the variables.

## Discussion

This case-control study describes is the first of its kind to describe intradialytic weight change as a factor associated with falls in dialysis patients. The study showed that patients in HD who suffer from falls have greater intradialytic weight changes. When patients arrive at the HD session, they are weighed by the nursing staff, who determine if there is excess weight relative to their reference weight or dry weight. The greater the weight difference, the greater the volume of liquid that should be removed. This usually occurs in patients who do not have good adherence to treatment or dietary guidelines^(20)^. The session lasts an average of 4 hours, so to remove a greater volume, a higher ultrafiltration rate must be used, which can generate a greater risk of intradialytic hypotension^(21)^ and orthostatic hypotension^(22)^. Previous studies have related these events with falls, and this study directly demonstrated that intradialytic weight change is a factor related to the risk of falls. Falls are factors of poor prognosis in patients who receive dialysis^(14)^ and after observing an incidence of falls of 32%, similar to the 37% described in a recent study^(23)^, it would be interesting to analyze the ability to prevent falls in patients whose intradialytic weight change is greater than 1.9 kg via prevention protocols.

Based on the other results of this study, patients receiving dialysis who fall also had significantly lower heart rates after HD than the controls. Physiologically, heart rate is supposed to increase when volemia decreases. If this does not occur, brain perfusion may be affected. The decrease in cardiac stimulus may be due to patients’ medication and in fact, this study found that patients receiving HD and who fall are more often treated with beta-blockers, which is consistent with the fact that these drugs are included into the group of those that increase the risk of falls^(24)^. However, the association between postHD heart rate and beta-blocker treatment was not statistically significant, so this hypothesis was ruled out. It seems likely that patients with a limited chronotropic response due to intrinsic or extrinsic causes are at greater risk of falls when undergoing HD.

As for the other drugs analyzed here, all belonging to the group of medications that increase the risk of falls, it was observed that patients who fell took antihypertensives more frequently than those in the control group. These findings are based on the association between lower preHD blood pressure values and increased risk of hypotension^(25)^ and falls^(26)^.

Regarding laboratory values, dialysis patients with a history of falls had higher levels of beta-2 microglobulin. Increased levels of this protein are due to the passing of time in renal replacement therapy: after 15 years receiving HD, about 80% of patients present with dialysis-related amyloidosis^(10)^, which can affect structures related to motor ability such as joints or the central nervous system. In our study, the involvement of these structures was not assessed since they had not been systematically analyzed and were not included in the patients’ medical history. The relationship between amyloid deposits and falls has been observed in patients with Parkinson’s disease^(27)^. However, to date, a relationship between falls and elevated beta-2 microglobulin or dialysis-associated amyloidosis levels had not been described, at least not in the studies we found. For this reason, we believe that it is necessary to conduct further prospective studies to acquire in-depth knowledge about this relationship.

On comparing the results of the balance tests of the cases and controls, patients who had suffered falls presented greater preHD instability than controls. This preHD alteration was observed in the anteroposterior direction and presented significantly higher values in terms of the range and velocity of movement of the center of pressure (CP). These results are similar to those found by other studies that analyzed patient balance in between dialysis sessions. A recent study found that higher velocity of CP movement was associated with falls^(28)^; in the present study, patients in the case group also had higher velocity of CP movement, but specifically in anteroposterior displacements. A previous study found a similar result, describing anteroposterior velocity as a fundamental stabilometric variable related to increased risk of falls^(29)^.

After dialysis, both cases and controls experienced an increase in the ranges, speeds and area of displacement of the CP, indicating the acute effect of HD on postural balance. This effect of HD was consistent with the results of other study^(9)^ and it used a similar methodology. Unlike another study^(18)^ which observed an increase in postHD lateral range associated with a higher risk of falls, in our study, we found no significant differences between cases and controls in postHD range and speed of CP movement, although the recorded instability remained higher among patients who had suffered falls. The fact that the increased postHD instability recorded among cases was not statistically significant for any of the stabilometric variables makes us think that the alterations that can result in falls do not improve in the periods between sessions. For this reason, future studies should determine the circumstances of falls, and whether these patients present hemodynamic or biochemical alterations in their daily lives more frequently.

The results shown here can help underpin a review of disconnection and discharge protocols of dialysis sessions, which are the responsibility of a HD unit’s nursing staff. This line of research could help test protection and surveillance measures for at-risk patients and verify their effectiveness in order to avoid falls and their consequences.

Limitations of this study include mainly its retrospective methodology. The time between assessments and the fall that defined the cases varied within the 6-month period considered in this study. Furthermore, the fall that defined a case was always outside the hospital and in the period between sessions, but the circumstances and the exact time of the falls were not taken into account.

## Conclusion

This case-control study identified some factors that dialysis unit nurses can pay attention to in order to reduce the postHD incidence of falls. Patients treated with HD who suffer falls are usually hypertensive, take antihypertensives [angiotensin-converting enzyme inhibitor (ACEI) or angiotensin II receptor antagonist (ARA II)] and beta-blockers, have elevated serum beta-2 levels microglobulin, and present anteroposterior instability. Unlike the controls, the patients who had a history of falls presented greater intradialytic weight change. Furthermore, the cases also presented lateral instability and a lower heart rate at the end of dialysis than the controls.
